# Genetic Drift, Purifying Selection and Vector Genotype Shape Dengue Virus Intra-host Genetic Diversity in Mosquitoes

**DOI:** 10.1371/journal.pgen.1006111

**Published:** 2016-06-15

**Authors:** Sebastian Lequime, Albin Fontaine, Meriadeg Ar Gouilh, Isabelle Moltini-Conclois, Louis Lambrechts

**Affiliations:** 1 Insect-Virus Interactions Group, Department of Genomes and Genetics, Institut Pasteur, Paris, France; 2 Centre National de la Recherche Scientifique, Unité de Recherche Associée 3012, Paris, France; 3 Université Pierre et Marie Curie, Cellule Pasteur UPMC, Paris, France; 4 Equipe Résidente de Recherche d’Infectiologie Tropicale, Division Expertise, Institut de Recherche Biomédicale des Armées, Brétigny-sur-Orge, France; 5 Unité Environnement et Risques Infectieux, Cellule d’Intervention Biologique d’Urgence, Department of Infection and Epidemiology, Institut Pasteur, Paris, France; 6 EA4655, Unité Risques Microbiens U2RM, Université de Caen Normandie, Caen, France; Fred Hutchinson Cancer Research Center, UNITED STATES

## Abstract

Due to their error-prone replication, RNA viruses typically exist as a diverse population of closely related genomes, which is considered critical for their fitness and adaptive potential. Intra-host demographic fluctuations that stochastically reduce the effective size of viral populations are a challenge to maintaining genetic diversity during systemic host infection. Arthropod-borne viruses (arboviruses) traverse several anatomical barriers during infection of their arthropod vectors that are believed to impose population bottlenecks. These anatomical barriers have been associated with both maintenance of arboviral genetic diversity and alteration of the variant repertoire. Whether these patterns result from stochastic sampling (genetic drift) rather than natural selection, and/or from the influence of vector genetic heterogeneity has not been elucidated. Here, we used deep sequencing of full-length viral genomes to monitor the intra-host evolution of a wild-type dengue virus isolate during infection of several mosquito genetic backgrounds. We estimated a bottleneck size ranging from 5 to 42 founding viral genomes at initial midgut infection, irrespective of mosquito genotype, resulting in stochastic reshuffling of the variant repertoire. The observed level of genetic diversity increased following initial midgut infection but significantly differed between mosquito genetic backgrounds despite a similar initial bottleneck size. Natural selection was predominantly negative (purifying) during viral population expansion. Taken together, our results indicate that dengue virus intra-host genetic diversity in the mosquito vector is shaped by genetic drift and purifying selection, and point to a novel role for vector genetic factors in the genetic breadth of virus populations during infection. Identifying the evolutionary forces acting on arboviral populations within their arthropod vector provides novel insights into arbovirus evolution.

## Author Summary

During infection of their arthropod vectors, arthropod-borne viruses (arboviruses) such as dengue viruses traverse several anatomical barriers that are believed to cause dramatic reductions in population size. Such population bottlenecks challenge the maintenance of viral genetic diversity, which is considered critical for fitness and adaptability of arboviruses. Anatomical barriers in the vector were previously associated with both maintenance of arboviral genetic diversity and alteration of the variant repertoire. However, the relative role of random processes and natural selection, and the influence of vector genetic heterogeneity have not been elucidated. In this study, we used high-throughput sequencing to monitor dengue virus genetic diversity during infection of several genetic backgrounds of their mosquito vector. Our results show that initial infection of the vector is randomly founded by only a few tens of individual virus genomes. The overall level of viral genetic diversity generated during infection was predominantly under purifying selection but differed significantly between mosquito genetic backgrounds. Thus, in addition to random evolutionary forces and the purging of deleterious mutations that shape dengue virus genetic diversity during vector infection, our results also point to a novel role for vector genetic factors in the genetic breadth of virus populations.

## Introduction

Due to the low fidelity of their RNA-dependent RNA polymerase, rapid replication kinetics and large population size, RNA viruses consist of a heterogeneous intra-host population of related mutants, sometimes referred to as a quasispecies [1]. This mutant swarm as a whole defines the properties of the viral population, and is considered critical for the fitness and adaptive potential of RNA viruses [1]. For example, high fidelity poliovirus mutants are attenuated in mice *in vivo*, demonstrating the functional importance of intra-host genetic diversity for pathogenesis [2].

Arthropod-borne viruses (arboviruses) are maintained by transmission between vertebrate hosts and blood-feeding arthropods such as mosquitoes that serve as vectors. Although arboviruses span a wide range of viral taxa in the *Togaviridae*, *Flaviviridae*, *Bunyaviridae*, *Rhabdoviridae* and *Orthomyxoviridae* families, the vast majority are RNA viruses, with the single known exception of a DNA arbovirus (African swine fever virus). The genetic plasticity of an RNA genome may confer arboviruses the remarkable ability to alternate between two fundamentally different hosts, and to quickly adapt to novel hosts [3]. Like other RNA viruses, high levels of intra-host genetic diversity are critical for arboviral fitness, as demonstrated in both host types for chikungunya virus [4,5] and West Nile virus [6–8].

Arboviruses usually rely on horizontal transmission between vertebrate hosts and arthropod vectors, although vertical transmission from an infected female arthropod to her offspring may occur [9,10]. After being ingested in a blood meal taken from a viremic vertebrate, arboviruses initially establish infection in the midgut epithelial cells of the arthropod vector. Transmission to another vertebrate host occurs after an extrinsic incubation period during which the arthropod develops a systemic infection that results in the release of viral particles in saliva. During infection of the arthropod vector, arboviruses are confronted with several anatomical barriers that are believed to impose severe population bottlenecks on viral populations [11]. Bottlenecks are dramatic reductions in population size, resulting in stochastic sampling of a small number of viral genomes from the mutant swarm. Population bottlenecks can significantly reduce the fitness of RNA viruses through accumulation of deleterious mutations that cannot be efficiently removed by purifying selection [12]. Initial infection and traversal of midgut cells, followed by virus dissemination and invasion of the salivary glands are expected to result in strong population drops that represents a challenge to maintaining arboviral genetic diversity during systemic vector infection [13].

Despite such population bottlenecks, arboviruses typically maintain high levels of genetic diversity during transmission by their arthropod vectors [11]. For example, analyses of West Nile virus populations in the midgut, hemolymph and saliva of *Culex* mosquitoes failed to document reductions in genetic diversity [14]. However, the authors of this study did not determine whether a large effective population size was maintained, or if viral genetic diversity was quickly replenished by mutation and demographic expansion following population bottlenecks. In a recent study of dengue virus (DENV), genetic diversity was maintained during human-to-mosquito transmission but the variant repertoire changed substantially between venous blood and different organs of *Aedes* mosquitoes that became infected by feeding on the person [15]. Over 90% of DENV genetic variants were lost upon transition from venous blood to mosquito abdomen, as well as from abdomen to salivary glands, which led the authors to estimate that about a hundred viral genomes initially established a productive midgut infection [15]. However, this number could have been underestimated because the calculation assumed that the observed change in variant frequency was due to chance alone (i.e., it did not account for the effect of natural selection). Genetic drift and purifying selection, for example, can result in a similar loss of genetic diversity.

The relative strength of natural selection and genetic drift is informed by the effective population size (*N*_*e*_), defined as the size of an idealized population that would drift at the same rate as the observed population [16]. *N*_*e*_ indicates whether the evolution of a population is better described as a deterministic (selection) or stochastic (drift) process. When *N*_*e*_ is large, competition between variants occurs with little interference of random processes. When *N*_*e*_ is small, stochastic sampling of variants counteracts selection and hinders adaptation. For example, genetic drift plays a limited role during systemic infection of the plant host by cauliflower mosaic virus, as viral populations maintain an effective size of several hundreds of viral genomes [17]. Understanding the relative role of genetic drift and natural selection is critical to evaluate the risk of arboviral emergence through adaptive processes [3]. For example, limited epidemic potential of an Asian lineage of chikungunya virus was associated with fixation of a deleterious deletion likely due to a founder effect [18].

In the present study, we investigated the intra-host evolution of DENV in the main mosquito vector *Aedes aegypti* by deep sequencing the full genome of viral populations at different time points of infection. Importantly, we accounted for the potential role of mosquito genetic variation on DENV intra-host genetic diversity. DENV intra-host genetic diversity has attracted considerable interest since the confirmation of its quasispecies nature [19]. Until now, however, most of this research has focused on viral genetic diversity in humans [20–23]. A few studies examined DENV intra-host genetic diversity in the mosquito vector [15,24,25], but these studies did not account for vector genetic heterogeneity. There is substantial evidence for genetic variation in *Ae*. *aegypti* vector competence for DENV [26–32], as well as specific interactions between *Ae*. *aegypti* genotypes and DENV genetic variants [33–37].

Our objectives were three-fold: (*i*) measure the bottleneck size during initial midgut infection of *Ae*. *aegypti* mosquitoes by DENV; (*ii*) monitor DENV intra-host genetic diversity during population expansion and systemic infection; and (*iii*) determine the influence of the vector genotype on bottleneck size and intra-host DENV genetic diversity.

## Material and Methods

### Ethics statement

The Institut Pasteur animal facility has received accreditation from the French Ministry of Agriculture to perform experiments on live animals in compliance with the French and European regulations on care and protection of laboratory animals. This study was approved by the Institutional Animal Care and Use Committee at Institut Pasteur.

### Virus and mosquitoes

This study used a wild-type DENV-1 isolate (KDH0026A) that was originally recovered from the serum of a clinically ill dengue patient attending Kamphaeng Phet Provincial Hospital, Thailand as previously described [36]. Informed consent of the patient was not necessary because the virus was isolated in laboratory cell culture for diagnostic purposes (unrelated to this study) and, therefore, was no longer a human sample. The isolate was passaged three times in *Aedes albopictus* C6/36 cells prior to its use in this study. The full-length consensus genome sequence of the isolate is available from GenBank under accession number HG316481.

*Aedes aegypti* females used in this study belonged to the 16^th^ generation of four isofemales lines (referred to as A, B, C, and D thereafter) derived from wild *Ae*. *aegypti* specimens collected in Kamphaeng Phet Province, Thailand. The lines were initiated by single mating pairs of field-caught males and females as previously described [36]. One male and one female from different collection sites (subdistricts) of the Muang district, Kamphaeng Phet Province, were randomly paired. The mothers of lines A and B, and the father of line C were collected in Thep Na Korn. The fathers of lines A, B and D were collected in Mae Na Ree. The mothers of lines C and D were collected in Nhong Ping Kai. They were maintained in the laboratory by mass sib-mating and collective oviposition at each subsequent generation. Quantification of genetic variation within and between the four isofemale lines was conducted as part of this study (see below).

To initiate the experiment, eggs were hatched in filtered tap water. Larvae were reared in 24×34×9 cm plastic trays at a density of about 200 larvae per tray. Adults were maintained in 30×30×30 cm screened cages under controlled insectary conditions (28±1°C, 75±5% relative humidity, 12:12 hour light-dark cycle). They were provided with cotton soaked in a 10% (m/v) sucrose solution *ad libitum* and allowed to mate for 6–7 days before the experimental infection.

### Restriction-site associated DNA (RAD) sequencing of mosquitoes

Genetic characterization of the *Ae*. *aegypti* isofemale lines used single nucleotide polymorphism (SNP) markers identified and genotyped by Restriction-site Associated DNA (RAD) sequencing [38]. Ten females from the 16^th^ generation of each isofemale line (i.e., from the same generation that was used in the experimental infection) and 10 females from the 1^st^ generation of an outbred population collected in 2013 in Thep Na Korn, Kamphaeng Phet Province, Thailand (i.e., the region where the isofemale lines originated) were genotyped using RAD sequencing.

Mosquito genomic DNA was purified using the procedure developed by Pat Roman's laboratory at the University of Toronto [39]. DNA concentration was measured with Qubit fluorometer and Quant-iT dsDNA Assay kit (Life Technologies, Paisley, UK). A modified version of the original double-digest Restriction-site Associated DNA (ddRAD) sequencing protocol [40] was used as previously described [41] with minor additional modifications. Briefly, 350 ng of genomic DNA from each mosquito were digested in a 50-μl reaction containing 50 units each of *NlaIII* and *MluCI* restriction enzymes (New England Biolabs, Herts, UK), 1× CutSmart Buffer and water for 3 hours at 37°C, without a heat-kill step. Digestion products were cleaned with 1.5× volume of Ampure XP paramagnetic beads (Beckman Coulter, Brea, CA, USA) and ligated to the modified Illumina P1 and P2 adapters with overhangs complementary to *NlaIII* and *MluCI* cutting sites, respectively. Each mosquito was uniquely labeled with a combination of P1 and P2 barcodes of variable lengths to increase library diversity at 5’ and 3’ ends (S1 Table). Ligation reactions were set up in a 45-μl volume with 2 μl of 4 μM P1 and 12 μM P2 adapters, 1,000 units of T4 ligase and 1× T4 buffer (New England Biolabs) and were incubated at 16°C overnight. Ligations were heat-inactivated at 65°C for 10 minutes and cooled down to room temperature (20–25°C) in a thermocycler at a rate of 1.5°C per 2 minutes. Adapter-ligated DNA fragments from all individuals were pooled and cleaned with 1.5× bead solution. Fragments from 350 to 440 base pairs (bp) were selected using a Pippin-Prep 2% gel cassette (Sage Sciences, Beverly, MA, USA). Finally, 1 μl of the size-selected DNA was used as a template in a 10-μl PCR reaction with 5 μl of Phusion High Fidelity 2× Master mix (New England Biolabs) and 1 μl of 50 μM P1 and P2 primers (S1 Table). To reduce bias due to PCR duplicates, 8 PCR reactions were run in parallel, pooled, and cleaned with a 0.8× bead solution to make the final library. At this step, final libraries were quantified by quantitative PCR using the QPCR NGS Library Quantification Kit (Agilent Technologies, Palo Alto, CA, USA).

Libraries containing multiplexed DNA fragments from 50 mosquitoes were sequenced on an Illumina NextSeq platform using a NextSeq 500 High Output 300 cycles v2 kit (Illumina, San Diego, CA, USA) to obtain 150-bp paired-end reads. An optimized final library concentration of 1.1 pM, spiked with 15% PhiX, was loaded onto the flow cell. Raw sequences were deposited in the NCBI Sequence Read Archive under accession number SRP075401.

### RAD markers for mosquito genotyping

A previously developed bash script [41] was used to process raw sequencing reads with minor modifications. Briefly, the DDemux program was used for demultiplexing fastq files according to the P1 and P2 barcodes combinations. Sequence quality scores were automatically converted into Sanger format. Sequences were filtered with FASTX-Toolkit. The first 5 bp (i.e., the restriction enzyme cutting site) and last 11 bp of P1 and P2 reads were trimmed. All reads with Phred scores <25 were discarded. P1 and P2 reads were then matched and unpaired reads were sorted as orphans.

Paired reads were aligned to the reference *Ae*. *aegypti* genome (AaegL3, February 2016) [42] using Bowtie version 0.12.7 [43]. Parameters for the ungapped alignment included a maximum of three mismatches allowed in the seed, suppression of alignments if more than one reportable alignment existed, and a “try-hard” option to find valid alignments. Orphans were combined with all unaligned paired reads and single-end alignment was attempted. All aligned Bowtie output files were merged per individual and were imported into the Stacks pipeline. A catalog of RAD loci used for SNP discovery was created using the ref_map.pl pipeline in Stacks version 1.37 [44,45]. First, sequences aligned to the same genomic location were stacked together and merged to form loci using Pstacks. Only loci with a sequencing depth ≥3X per individual were retained. Cstacks was used to create a catalog of consensus loci, merging alleles together and Sstacks was used to match all identified loci. The Stacks pipeline identified a total of 899,892 RAD loci. The “populations” module was used to export markers with a sequencing depth ≥10X that were present in ≥98% of samples. The mosquito phylogenetic analysis was performed with the resulting subset of 2,321 SNPs, which belonged to 1,319 distinct RAD loci (0.15%).

### Phylogenetic analysis of mosquitoes

Phylogenetic trees were constructed using a Bayesian Markov Chain Monte Carlo (MCMC) algorithm, implemented in the BEAST 1.8.3 package [46]. Inferences were produced under the coalescent model (constant size), and under the GTR+G (global time reversible with gamma distribution and no invariable sites) and the HKY+G (Hasegawa-Kishino-Yano) substitution models. Heterozygote positions were considered in calculations by enabling the use of IUPAC code and associated degeneracy within the substitution model. The length of MCMC was set at 3x10^7^ states to obtain Effective Sampling Size (ESS) values >200.

### Experimental mosquito infection

Six- to seven-day-old *Ae*. *aegypti* females were deprived of water and sucrose for 24h prior to the infectious blood meal. The virus stock was diluted in cell culture medium (Leibovitz’s L-15 medium + 10% heat-inactivated fetal calf serum + non-essential amino-acids + 0.1% penicillin/streptomycin + 1% sodium bicarbonate) to reach an expected infectious titer of 3×10^6^ focus-forming units (FFU) per mL. One volume of virus suspension was mixed with two volumes of freshly drawn rabbit erythrocytes washed in distilled phosphate-buffered saline (DPBS). After gentle mixing, 2.5 mL of the infectious blood meal was placed in each of several Hemotek membrane feeders (Hemotek Ltd, Blackburn, UK) maintained at 37°C and covered with a piece of desalted porcine intestine as a membrane. Sixty μL of 0.5 M ATP were added to each feeder as a phagostimulant. Each isofemale line was allowed to feed during two rounds of 15 min on different feeders to ensure randomization of a potential feeder effect. Actual virus titer in the blood meal was measured by standard focus-forming assay in C6/36 cells [33]. After feeding, mosquitoes were cold anesthetized on ice and fully engorged females were transferred to 1-pint cardboard cups. They were incubated under controlled conditions (28±1°C, 75±5% relative humidity, 12:12 hour light-dark cycle) in a climatic chamber.

At 4, 7 and 14 days post exposure (dpe), the midgut of 8–12 individuals from each isofemale line (i.e., biological replicates) were dissected. Midguts were homogenized individually in 140 μL of DPBS + 560 μL of QIAamp Viral RNA Mini Kit lysis buffer (Qiagen, Hilden, Germany) during two rounds of 30 sec at 5,000 rpm in a mixer mill (Precellys 24, Bertin Technologies, Montigny le Bretonneux, France). At 7 and 14 dpe, the legs of midgut-dissected mosquitoes were removed and homogenized as described above. At 14 dpe, the salivary glands of the midgut- and leg-less individuals were harvested and processed as above.

### Virus deep sequencing

Total RNA was extracted from mosquito homogenates using QIAamp Viral RNA Mini Kit (Qiagen) and reverse transcribed using Transcriptor High Fidelity cDNA Synthesis Kit (Roche Applied Science, Penzberg, Germany) and a specific reverse primer located at the 3’ end of the viral genome (S1 Table). Presence and amount of viral cDNA was assessed by quantitative PCR using the LightCycler DNA Master SyberGreen I kit (Roche Applied Science) and custom primer pairs (S1 Table). Absolute quantification used a standard curve generated with serial dilutions of PCR amplicons of known concentration. Selected samples were amplified by 40 cycles of PCR in 10 overlapping amplicons with Q5 High Fidelity DNA polymerase (New England Biolabs) and custom primer pairs (S1 Table).

PCR products were purified with Agencourt AMPure XP magnetic beads (Beckman Coulter) and their concentration was measured by Quant-iT PicoGreen dsDNA fluorometric quantification (Invitrogen). Equal amounts of each amplicon were pooled by sample and brought to a final concentration of 0.2 ng/μL. Multiplexed libraries were prepared using Nextera XT DNA Library Preparation Kit (Illumina) and single-end sequenced on an Illumina NextSeq 500 platform using a high-output 75 cycles v1 kit (Illumina). Sequencing reads were demultiplexed using bcl2fastq v2.15.0 (Illumina). Raw sequences were deposited in the NCBI Sequence Read Archive under accession number SRP075335.

After demultiplexing, reads were trimmed to remove Illumina adaptor sequences using Trimmomatic v0.33 [47] and amplification primers if matching sequences were found on either the 5’ or 3’ end of the reads using Cutadapt v.1.8.3 [48]. Reads shorter than 32 bp were discarded and remaining reads were then mapped to the reference DENV genome sequence using Bowtie2 v2.1.0 [49]. The alignment file was converted, sorted and indexed using Samtools v0.1.19 [50]. Coverage and sequencing depth were assessed using bedtools v2.17.0 [51]. Single nucleotide variants (SNVs) and their proportion among all reads were called using LoFreq* v2.1.1 [52] and their effect at the amino-acid level assessed by SNPdat v.1.0.5 [53].

### Viral genetic diversity analyses

Two sets of SNV markers were used for analyses of genetic diversity and natural selection. The ‘full’ marker set excluded all nucleotide positions in a given sample that had (*i*) a sequencing depth <500X or (*ii*) where potential sequencing or library preparation artifacts [54] were detected. The ‘conservative’ marker set excluded all nucleotide positions in all samples that had (*i*) a sequencing depth <500X or (*ii*) where potential sequencing or library preparation artifacts [54] were detected in a least one sample. The conservative marker set minimized the potential bias owing to the unique mutational profile of each nucleotide position. However, because some of the overlapping fragments covering the viral genome could not be successfully amplified in a few samples (S1 Fig), the conservative marker set failed to cover large fractions of the viral genome (S2A Fig). The full marker set, conversely, minimized the potential bias owing to distinct evolutionary properties of the different regions of the viral genome.

Genetic complexity of the viral population was estimated using normalized Shannon entropy (*S*_*n*_) for each nucleotide site [55]:

$$S_{n}\;=\;\frac{-(p\text{ ln}(p))+((1-p)\times \text{ln}(1-p))}{ln(4)}$$

where *p* is the SNV minor allele frequency at the considered position, and ln(4) corresponds to maximum complexity (i.e., four possible nucleotides at each position). For individual SNVs, *S*_*n*_ values range from 0 to 1. For diallelic SNVs, *S*_*n*_ values range from 0 (no diversity) to 0.5 (maximum complexity, when the two alternative nucleotides are present at equal frequency). For each sample, *S*_*n*_ was averaged over all nucleotide sites included in either the full or the conservative set of SNV markers (i.e., total genome length minus number of excluded positions).

Genetic diversity of the viral population was also estimated using nucleotide diversity at each nucleotide site [56]:

$$\pi \;=\;\frac{D}{D-1}\;\times \;(1-(p^{2}+{\left(p-1\right)}^{2})$$

where *D* is the sequencing depth at the considered position and *p* is the SNV minor allele frequency. Like for *S*_*n*_, *π* values for a diallelic SNV range from 0 (no polymorphism) to 0.5 (when the two alternative nucleotides are present at equal frequency). For each sample, *π* was averaged over all nucleotide sites included in either the full or the conservative set of markers. This index of genetic diversity is less sensitive to low-frequency variants than *S*_*n*_, due to the lack of log-transformation of the frequencies.

### Natural selection assessment

The magnitude and direction of natural selection were assessed using the *d*_*N*_/*d*_*S*_ ratio, which is the ratio between the number of non-synonymous substitutions per non-synonymous site (*d*_*N*_) over the number of synonymous substitutions per synonymous site (*d*_*S*_) of a coding sequence, assuming synonymous substitutions are selectively neutral:

$$d_{S}\;=\;\frac{-3\;\times \text{ln}\left(1-\frac{4\;\times \frac{S_{d}}{S_{s}}}{3}\right)}{4}{\;and\;d}_{N}\;=\;\frac{-3\;\times \text{ln}\left(1-\frac{4\;\times \frac{N_{d}}{N_{s}}}{3}\right)}{4}$$

where *S*_*d*_ is the number of synonymous substitutions in the sequence, *S*_*s*_ is the number of synonymous sites, *N*_*d*_ is the number of non-synonymous substitutions in the sequence and *N*_*s*_ is the number of non-synonymous sites [57]. A *d*_*N*_/*d*_*S*_ ratio >1 means that there is an excess of normalized number of non-synonymous substitutions relative to the normalized number of synonymous substitutions and is interpreted as evidence for positive selection (i.e., driving change). A *d*_*N*_/*d*_*S*_ ratio <1 means that there is an excess of normalized number of synonymous substitutions relative to the normalized number of non-synonymous substitutions and is interpreted as evidence for negative selection (i.e., acting against change). A *d*_*N*_/*d*_*S*_ ratio equal to 1 is interpreted as evidence for the absence of natural selection (i.e, neutral evolution).

The *d*_*N*_/*d*_*S*_ ratio was computed using the Nei-Gojobori method [57] with suggested modifications for high-throughput sequencing data [58]. Briefly, *N*_*d*_ and *S*_*d*_ were calculated for each sample as the sum of SNV frequencies. Mean *N*_*d*_ and *S*_*d*_ were computed for each isofemale line at each time point and used for *d*_*N*_ and *d*_*S*_ calculation, respectively. Numbers of synonymous and non-synonymous sites from the initial population consensus sequence were estimated using MEGA v.7.0.16 [59] by computing the number of 0-, 2-, 3- and 4-fold degenerate sites following the Nei-Gojobori method [57]. The full marker set had a variable number of synonymous and non-synonymous sites depending of the number of nucleotide sites retained or excluded for each sample. The conservative marker set had 328.67 synonymous and 1,481.33 non-synonymous sites for all samples.

### Statistical testing

Statistical analyses were performed in the statistical environment R, version 3.2.0 (http://www.r-project.org/) using the packages car [60], plyr [61], ggplot2 [62], stringr [63], reshape2 [64], gridExtra [65], fitdistrplus [66] and boot [67]. In all analyses, the individual mosquito sample was considered a biological unit of replication.

Infection prevalence and cDNA copy numbers were compared among isofemale lines at each time point by pairwise Pearson χ^2^ tests and pairwise Wilcoxon signed-rank tests, respectively, followed by a Holm correction of *p*-values for multiple testing.

The proportion of SNVs per position, mean *S*_*n*_ and mean *π* estimates were compared between the input and later time points using pairwise Wilcoxon signed-rank tests and a Holm *p*-value adjustment. The proportion of SNVs per position, *S*_*n*_, *π* and *d*_*N*_/*d*_*S*_ estimates in midgut samples were analyzed between 4 and 7 dpe as a function of the combined effects of time point and mosquito genotype using a linear model with an identity link function and a normal error distribution. Model validity was verified with quantile-quantile (Q-Q) plots of residuals and by computing Cook’s distance to assess influence of observations. Statistically significant effects (*p*<0.05) of time point, mosquito genotype and their interactions were determined using type-II analysis of variance. Statistically insignificant interactions were removed from the model, subsequently repeating model validation. Statistical testing of pairwise differences between isofemale lines used the linear regression coefficients. Estimated regression coefficients were extracted and their 95% confidence intervals and *p*-values were calculated based on their standard errors compared to a reference level. Isofemale line A was arbitrarily chosen as the reference level.

### Bottleneck size estimation

Following a published method [17], bottleneck size at initial midgut infection was estimated by analyzing the change in frequency distribution of neutral markers between blood meal (initial) and midgut (final) samples. Under the assumption of neutrality (i.e., absence of natural selection), the idealized number of founding genomes (*N*) initiating the midgut infection can be computed as:

$$N\;=\;\frac{p(1-p)}{Var(p^{\prime })-Var(p)}$$

where *p* is the marker allele frequency in the initial population and *p′* is the marker allele frequency in the final population [17]. This method considers that changes in the genetic variance between sequential samples result exclusively from genetic drift and therefore requires neutral or quasi-neutral markers.

SNVs that were presumably neutral were selected based on the following set of criteria: (*i*) synonymous change at the third codon position, (*ii*) no significant change in mean frequency between sampling time points, (*iii*) SNV detected in ≥80% of the five viral input replicates (viral stock and blood meal samples), and (*iv*) mean frequency >0.02 in the input population. Confidence intervals of *N* estimates were computed using a bootstrapping procedure as described in [17]. Briefly, for each bootstrap all individuals were sampled with replacement to calculate *N*. This was repeated 1,000 times to generate a distribution of *N* values and derive 95% confidence intervals corresponding to the 2.5 and 97.5 percentiles of the distribution.

### Bottleneck simulation

The effect of the initial midgut infection bottleneck on viral diversity indices was simulated in R based on 100 sampling events from an initial viral population containing 100 independent SNVs. SNV minor allele frequency was randomly drawn from an exponential distribution (*λ* = 100). Initial viral population size (equivalent to the infectious dose ingested in the blood meal) was drawn from a normal distribution (mean = 2,000; standard deviation = 200). Bottleneck size was drawn from a normal distribution (mean = 28; standard deviation = 5). Mean *S*_*n*_ and mean *π* were computed for all samples in the presence or the absence of a detection threshold arbitrarily set at an SNV minor allele frequency of 0.01.

## Results

### Mosquito genetic variation

**Fig 1:**
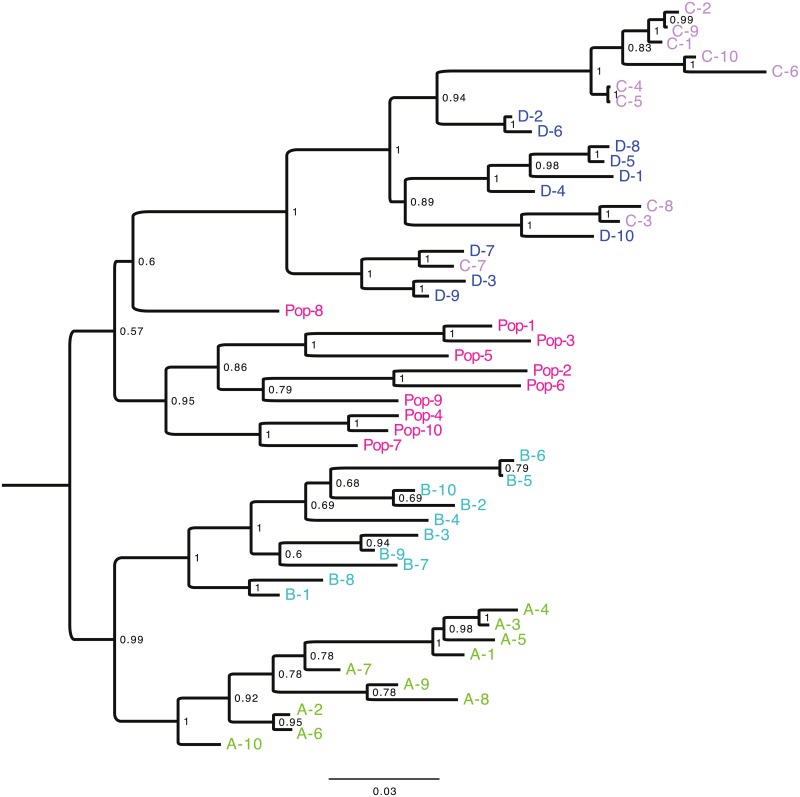
Phylogenetic relationships between *Aedes aegypti* isofemale lines. Bayesian phylogenetic tree representing the genetic diversity across individuals from the four isofemale lines (A, B, C and D) and from a field-derived outbred population (Pop) from the same geographic location where the lines were created. The phylogenetic analysis was based on a GTR+G substitution model of 2,321 SNPs. Putative populations are depicted in different colors. The scale bar indicates the number of substitutions and posterior probabilities are displayed at relevant nodes.

A genome-wide set of 2,321 SNPs generated by RAD sequencing was used to genetically characterize the four *Ae*. *aegypti* isofemale lines (A, B, C, and D). These markers had a sequencing depth ≥10X per sample and were missing in <2% of samples. An outbred *Ae*. *aegypti* population from the same geographic location where the lines were created was also genotyped to provide a phylogenetic background. Phylogenetic relationships among individuals from the four isofemale lines and the outbred population were determined with a Bayesian method (Fig 1). As expected, the outbred mosquito population was paraphyletic, reflecting its genetic diversity. Mosquitoes from isofemale lines A and B clustered independently with strong statistical support, confirming their distinct genetic identity. Unexpectedly, mosquitoes from isofemale line C grouped with mosquitoes from isofemale line D within the same clade. This could be the result of relatedness between the parents randomly chosen to initiate the lines, as the mothers of lines C and D came from the same collection site and may have been siblings. Two different substitution models for the phylogenetic reconstruction gave similar clustering patterns. Similar results were also obtained when testing a variable number of markers (allowing from 0% to 30% of missing genotypes) with the same method. Because isofemale lines C and D were not unambiguously assigned to different monophyletic groups, they could not be considered as distinct genotypes and were thus combined in all subsequent analyses. They are jointly referred to as line CD hereafter.

### Infection time course and sample selection

**Fig 2:**
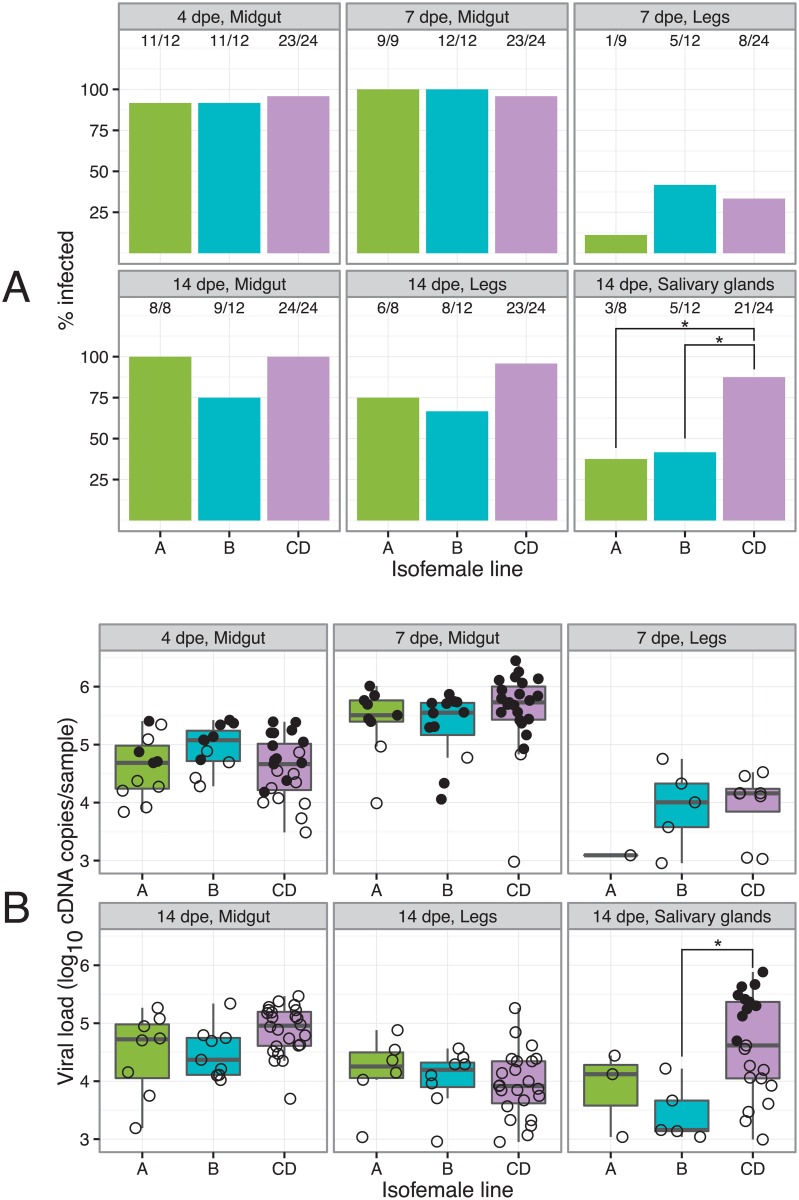
Time course of DENV prevalence and viral load. (A) Bar graphs show the percentage of DENV-infected samples stratified by time point, tissue and isofemale line. Relative numbers of positive samples are indicated above the bars. (B) Box plots show the number of DENV genome copies per infected sample stratified by time point, tissue and isofemale line. Solid dots represent individual samples selected for deep sequencing and open dots represent samples that were not sequenced. dpe = days post exposure. * *p*<0.05.

Mosquitoes from the three different genotypes (A, B, and CD) were exposed to DENV through an artificial blood meal at a final titer of 1.52×10^6^ focus-forming units (FFU)/mL. Assuming a blood meal size of approximately 2 μL, the infectious dose ingested by each mosquito was about 3,000 infectious viral particles. The proportion of mosquitoes that acquired a midgut infection ranged from 75 to 100% and did not differ significantly between time points or isofemale lines (Fig 2A). The proportion of mosquitoes with a DENV infection that disseminated to their legs increased from 10–40% at 7 days post exposure (dpe) to 60–100% at 14 dpe, but the rate of virus dissemination to the legs did not differ significantly between isofemale lines (Fig 2A). However, the proportion of mosquitoes with a disseminated infection in the salivary glands was significantly higher for line CD (87.5%) than for line A (37.5%) and line B (41.7%) at 14 dpe (line A vs. line CD, *p* = 0.037; line B vs. line CD, *p* = 0.037). Among infected mosquitoes, viral load ranged from 8.9×10^2^ to 2.8×10^6^ DENV genome copies per sample with no significant difference between isofemale lines at any of the time points, with the exception of lines B and CD that had significantly different viral loads (*p* = 0.037) in their salivary glands at 14 dpe (Fig 2B).

Deep sequencing of viral genomes was performed for a subset of 78 infected samples at selected time points (Fig 2B) that were processed individually and treated as biological replicates. Some samples were excluded because their low concentration of viral RNA resulted in unsuccessful RT-PCR amplification. A total of 4, 7 and 13 infected midguts at 4 dpe and 7, 11 and 21 infected midguts at 7 dpe were analyzed for lines A, B, and CD, respectively. Ten infected salivary glands at 14 dpe were analyzed in line CD. In addition, DENV genomes were deep sequenced in the initial viral stock and in four replicates of the infectious blood meal. On average, 3,615,466 sequencing reads per sample aligned to the reference DENV genome. Mean DENV genome coverage with a sequencing depth >500X was 10,594 nucleotides per sample, which represents 98.8% of the 10,718 nucleotides of the total genome length. Mean sequencing depth was 24,212X per sample (S1 Fig).

### Patterns of viral genetic diversity

The full set of SNV markers retained for population genetic analyses included an average of 5,843 nucleotide sites across the DENV genome, whereas a more conservative set (see Materials and Methods) was restricted to 1,810 nucleotides (S2A Fig). SNVs of the full marker set were randomly distributed across the genome without obvious mutation hot or cold spot (S3 Fig). A new variant reached consensus level (frequency >0.5) in one midgut sample at 4 dpe and one midgut sample at 7 dpe, but the SNV was different in each case. In salivary glands collected at 14 dpe, new variants reached consensus level at 11 positions, none of which was shared among individuals within or between isofemale lines (S3 Fig). In the more restricted conservative set of markers, no variant reached consensus level at any time point (S2B Fig).

**Fig 3:**
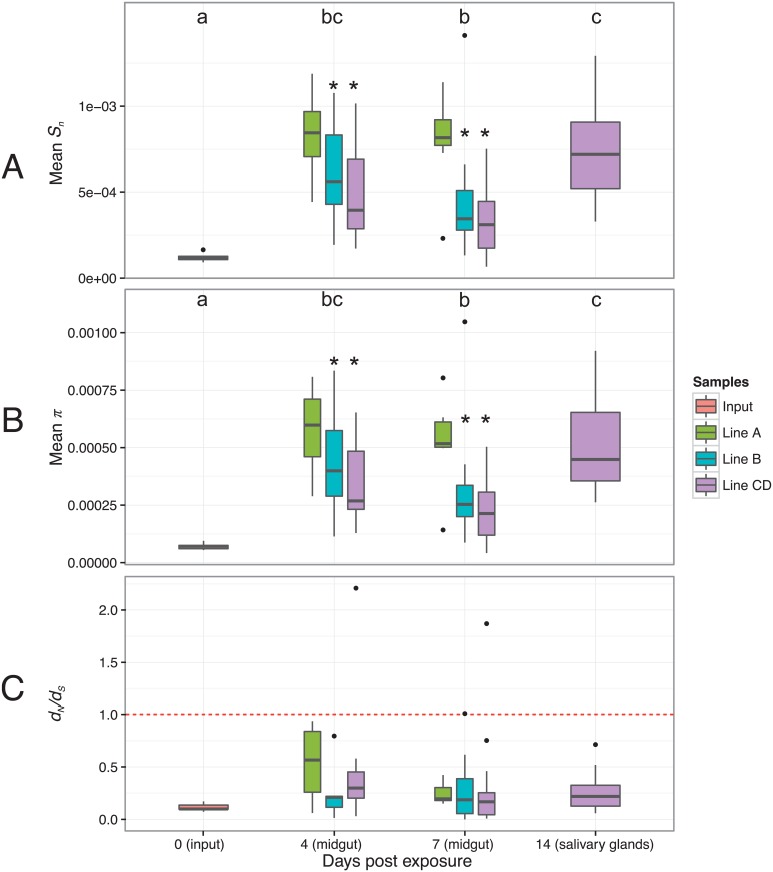
Observed levels of DENV intra-host genetic diversity and natural selection assessment. (A) Averaged Shannon entropy (*S*_*n*_) per site over all positions per sample. (B) Averaged nucleotide diversity (*π*) over all positions per sample. (C) *d*_*N*_/*d*_*S*_ ratios over all coding positions per sample. The horizontal, dashed red line represents a *d*_*N*_/*d*_*S*_ ratio of 1, which is interpreted as evidence for neutral evolution (i.e., absence of natural selection). A *d*_*N*_/*d*_*S*_ ratio >1 is interpreted as evidence for positive selection; a *d*_*N*_/*d*_*S*_ ratio <1 it is interpreted as evidence for negative (purifying) selection. Letters above the graph indicate statistically significant pairwise differences between time points. For midgut samples, stars above the bars indicate statistically significant pairwise differences between isofemale lines, with line A as the reference level.

To determine the effect of initial midgut infection on DENV genetic diversity, a first series of analyses compared viral genetic diversity observed in the input samples with genetic diversity observed at any of the later time points. In the full marker set, initial infection of the midgut was associated with an increase in viral genetic diversity relative to the input (Fig 3) both when measured with normalized Shannon entropy *S*_*n*_ (0 dpe *vs*. 4 dpe, *p* = 0.0001; 0 dpe *vs*. 7 dpe, *p* = 0.002; 0 dpe *vs*. 14 dpe, *p* = 0.003) (Fig 3A) and when measured with nucleotide diversity *π* (0 dpe *vs*. 4 dpe, *p* = 0.0001; 0 dpe *vs*. 7 dpe, *p* = 0.0004; 0 dpe *vs*. 14 dpe, *p* = 0.003) (Fig 3B). Viral diversity was also significantly higher in the salivary glands at 14 dpe than in the midgut at 7 dpe (*S*_*n*_: *p* = 0.012; *π*: *p* = 0.012). The proportion of variable sites detected also increased following initial midgut infection (S4 Fig) although differences were only statistically significant between 0 dpe and 4 dpe (*p* = 0.0029) and between 0 dpe and 14 dpe (*p* = 0.0067). Similarly, in the conservative set of markers, mosquito infection was associated with a relative increase in viral genetic diversity following initial midgut infection, albeit more modestly due to the smaller number of markers, both when measured with normalized Shannon entropy *S*_*n*_ (0 dpe *vs*. 4 dpe, *p* = 0.046; 0 dpe *vs*. 14 dpe, *p* = 0.008) (S5B Fig) and when measured with nucleotide diversity *π* (0 dpe *vs*. 14 dpe, *p* = 0.008) (S5C Fig). The proportion of variable sites detected, however, did not differ statistically between time points (S5A Fig).

To evaluate the dynamics of DENV genetic diversity during viral population expansion in the midgut, a second series of analyses compared viral genetic diversity between 4 and 7 dpe, accounting for potential differences between mosquito genotypes. In the full set of markers, both the time point (proportion of variable sites: *p* = 0.03; *S*_*n*_: *p* = 0.04; *π*: *p* = 0.04) and the isofemale line (proportion of variable sites: *p* = 0.0035; *S*_*n*_: *p* = 0.0002; *π*: *p* = 0.0005) significantly influenced viral genetic diversity. Overall, DENV genetic diversity slightly decreased between 4 dpe and 7 dpe. Isofemale line A displayed significantly higher viral genetic diversity than lines B and CD, for all three indices: proportion of variable sites (*p* = 0.012 and *p* = 0.0008, respectively), *S*_*n*_ (*p* = 0.006 and *p* = 0.0005 respectively) and *π* (*p* = 0.017 and *p* = 0.0001, respectively). Similar results were obtained with the conservative set of markers. Both the time point (proportion of variable sites: *p* = 0.01; *S*_*n*_: *p* = 0.013; *π*: *p* = 0.015) and the isofemale line (proportion of variable sites: *p* = 0.0011; *S*_*n*_: *p* = 0.0006; *π*: *p* = 0.0029) significantly influenced viral genetic diversity. Overall, DENV genetic diversity slightly decreased between 4 dpe and 7 dpe. Isofemale line A displayed significantly higher viral genetic diversity than lines B and CD, for all three indices: proportion of variable sites (*p* = 0.017 and *p* = 0.0002, respectively), *S*_*n*_ (*p* = 0.0047 and *p* = 0.0001, respectively) and *π* (*p* = 0.012 and *p* = 0.0007, respectively).

### Natural selection

Based on the full set of SNV markers, *d*_*N*_/*d*_*S*_ ratios were predominantly negative indicating purifying selection (Fig 3C). There was no statistically significant difference in *d*_*N*_/*d*_*S*_ ratios between time points or mosquito isofemale lines. Computing *d*_*N*_/*d*_*S*_ ratios was not possible with the conservative set of markers because the smaller number of SNVs resulted in a large proportion of samples with *d*_*S*_ = 0. Analysis of *d*_*N*_/*d*_*S*_ ratios calculated per isofemale line, however, provided results consistent with predominantly purifying selection using the conservative set of markers. Average *d*_*N*_/*d*_*S*_ ratios were remarkably similar among lines and time points around 0.2218 (S1 Table).

### Bottleneck size estimates

**Fig 4:**
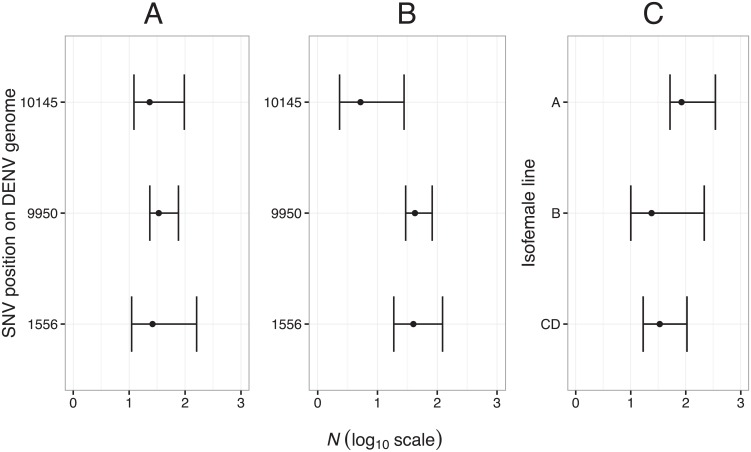
Estimates of bottleneck size at initial midgut infection. The estimated number of founding genomes (*N*) that contribute to initial midgut infection is shown for three markers identified by their position on the DENV genome (1556, 9950, 10145). The three markers are SNVs that are assumed to evolve neutrally or quasi-neutrally. Horizontal bars indicate confidence intervals of *N* estimates computed by bootstrapping. (A) *N* estimates based on samples collected at 4 dpe. (B) *N* estimates based on samples collected at 7 dpe. (C) *N* estimates for each isofemale line obtained for marker 1556 using combined 4 dpe and 7 dpe samples.

**Fig 5:**
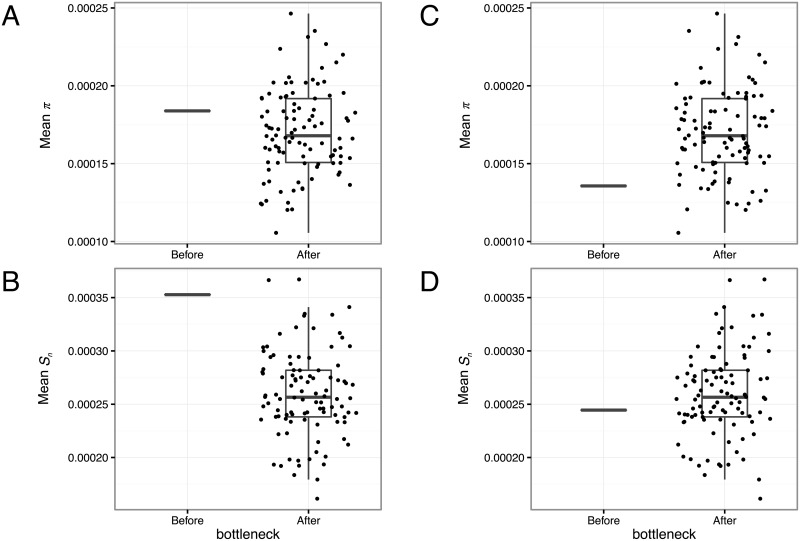
Simulated effects of a population bottleneck and SNV detection threshold on observed levels of genetic diversity. The simulation considered 100 SNVs present in the input population, which were sampled 100 times (infection events) with randomly assigned SNV frequency, initial viral population size and bottleneck size. In the population sampled following the bottleneck, mean *π* (A, C) and *S*_*n*_ (B, D) are shown when no frequency detection threshold was set (A, B), and when a 1% frequency detection threshold was set (C, D).

Three SNVs that complied with criteria of quasi-neutral evolution were selected to estimate the idealized number of founding viral genomes (*N*) initiating the midgut infection based on changes in the variance of their frequency between input and midgut samples (Table 1). Based on the three markers, initial midgut infection was founded by 23–34 genomes when estimated at 4 dpe (Fig 4A) and 5–42 genomes when estimated at 7 dpe (Fig 4B). Collectively, 95% confidence intervals ranged from 2 to 161 founding genomes. *N* estimates and their confidence intervals were consistent across time points, especially for marker at position 1556. For this marker, 4 dpe and 7 dpe data were pooled to compute isofemale line-specific *N* estimates. There were no statistically significant differences among lines in the estimated bottleneck size (Fig 4C), ranging from 83 (95% confidence interval: 52–396) founding genomes for line A, to 23 (9–220) for line B and 33 (16–108) for line CD.

**Table 1:** SNV markers used for bottleneck size estimation.

SNV position	Mutation	Position in codon	Amino acid	Viral gene	Initial frequency (mean ± sd)	Final frequency 4 dpe (mean ± sd)	Final frequency 7 dpe (mean ± sd)
1556	A → C	3^rd^	L	E	0.021 ± 0.004	0.026 ± 0.028	0.031 ± 0.023
9950	C → A	3^rd^	T	NS5	0.117 ± 0.022	0.108 ± 0.059	0.095 ± 0.054
10145	C → T	3^rd^	T	NS5	0.051 ± 0.011	0.021 ± 0.047	0.043 ± 0.097

dpe = days post exposure; sd = standard deviation.

### Bottleneck simulations

Simulations were performed to model the effect of population bottlenecks on DENV intra-host genetic diversity. The simulation randomly assigned SNV minor allele frequency, initial viral population size and bottleneck size to explore whether a minimum threshold for SNV detection would alter the observed genetic diversity following a population bottleneck compared to the true genetic diversity. When 100 SNVs were present in the input viral population and no minimum detection threshold was set, mean *S*_*n*_ and *π* estimated in 100 replicate samples decreased following the bottleneck (Fig 5A). However, when only SNVs with a minor allele frequency >1% were detected, mean *S*_*n*_ and *π* estimates increased after the bottleneck (Fig 5B).

## Discussion

We investigated the evolutionary forces acting on DENV populations within their arthropod vector. Specifically, we evaluated the relative effects of natural selection and genetic drift on DENV intra-host evolution in the midgut of *Ae*. *aegypti*. In addition, we assessed the influence of vector genetic heterogeneity on intra-host viral genetic diversity. Our results show that DENV intra-host genetic diversity in *Ae*. *aegypti* is shaped by the combined effects of genetic drift, purifying selection and vector genotype. Reshuffling of the variant repertoire during initial infection of the midgut was associated with a bottleneck size ranging from 5 to 42 founding viral genomes, irrespective of the mosquito genotype. DENV genetic diversity increased significantly following initial infection, but was restricted by strong purifying selection during DENV population expansion in the midgut. Observed levels of DENV genetic diversity in the midgut differed significantly between mosquito isofemale lines despite a similar bottleneck size at initial infection.

Arboviruses typically maintain high levels of genetic diversity during transmission by their arthropod vectors despite anatomical barriers that often result in severe population drops [11]. Such population bottlenecks have been documented for several arboviruses in their vectors using artificial mutant swarms [68], marked viral clones [13], viral replicons [69] or stochastic simulations based on observed changes in variant frequencies [15]. Although the overall level of arboviral genetic diversity is usually maintained during vector infection [14], the viral variant repertoire can be significantly altered [15,68,70]. Presumably, viral genetic diversity is quickly replenished by mutation and demographic expansion following population bottlenecks [11]. However, whether changes in the viral variant repertoire are due to stochastic sampling (i.e., genetic drift), purifying selection (i.e., removal of variants with lower fitness), or vector genetic heterogeneity combined with specific interactions between vector and virus genotypes [33–37] has remained largely unresolved. Our analysis used neutral or quasi-neutral genetic markers to estimate the effective DENV population size during initial infection of the *Ae*. *aegypti* midgut. This approach rules out natural selection and only measures the effect of genetic drift due to random sampling. It is worth noting, however, that true neutral mutation may not exist because even synonymous mutations can have a fitness effect, especially in RNA viruses [71]. Deviation from our assumption of neutrality or quasi-neutrality of the chosen markers may have overestimated the bottleneck size. Indeed, both positive selection and negative selection would likely act to decrease the variance of marker frequency and therefore result in a larger estimate of *N*_*e*_ with our method. Therefore, our conclusion that DENV populations undergo a strong population bottleneck during initial midgut infection should be robust to any undetected departure from neutrality. Moreover, we chose markers whose average frequency was similar before and after the bottleneck, supporting the assumption that they were not under directional selection. Our estimation that initial midgut infection is founded by only a few tens of DENV genomes is consistent with previous estimations for DENV based on stochastic simulations using empirical data [15]. We went one step further by demonstrating that genetic drift, rather than natural selection, is the main evolutionary force underlying this population bottleneck.

Although our estimated bottleneck size is larger than for other RNA viruses during host-to-host transmission [72], it is expected to substantially reduce the genetic breadth of the viral quasispecies [73]. This finding has important implications for DENV evolution in general. A small effective population size means that natural selection will be relatively inefficient during human-to-mosquito transmission. It will prevent adaptive evolution especially if beneficial SNVs are present at low frequencies in the mutant swarm transmitted from the human host [74]. On the other hand, the population bottleneck associated with initial midgut infection may not be small enough to prevent the long-term maintenance of defective viral genomes through complementation by co-infection of host cells with functional viruses. Such a phenomenon was previously documented in the case of a stop-codon mutation that became widespread in DENV populations sampled in Myanmar in 2001 [75]. The frequency of the stop-codon mutation was likely high enough to overcome the effect of population bottlenecks during multiple host-to-host transmission events.

During DENV population expansion following initial midgut infection, natural selection was predominantly negative (i.e., acting against change). Accordingly, the consensus sequence remained unchanged in most of the midgut samples. Only in the salivary glands did several SNVs reach consensus level (frequency >0.5), but with no evidence of evolutionary convergence. As was observed for West Nile virus [68], DENV intra-host genetic diversity in midguts slightly decreased between 4 and 7 dpe. Importantly, we found that overall levels of DENV intra-host genetic diversity differed significantly between distinct mosquito genetic backgrounds. Both the initial bottleneck size and the census size of the viral population did not significantly vary among mosquito genotypes, and thus are unlikely to explain this difference. The mechanistic basis of this finding remains to be determined, but we speculate that viral populations may undergo different selective constraints in different mosquito genotypes. Mosquito genotypes could vary in the intensity of purifying selection (i.e., variation in the efficiency of mechanisms that remove deleterious *de novo* mutations), but this was not supported by our data. Likewise, the overall lack of positive selection that we observed indicates that it is unlikely to be the underlying mechanism. Alternatively, mosquito genotypes may differ in the level of balancing selection (i.e., mechanisms that act to promote genetic diversity such as negative frequency-dependent selection or spatiotemporal fluctuations in the strength and direction of selection). The antiviral RNA interference (RNAi) pathway of mosquitoes was suggested to play a role in viral genetic ‘diversification’ [76,77], by promoting escape to complementary base-pairing required for RNAi-mediated cleavage. Variation in host factors could also result in differences in viral intra-host genetic diversity through subtle changes in viral RNA-dependent RNA polymerase fidelity [78]. Mutation rates of RNA viruses are not only determined by virus-encoded factors, by also by host-dependent processes. Replicase fidelity of a plant RNA virus was found to differ according to the host type [79]. Replication fidelity in retroviruses can be affected by intracellular dNTP imbalance [80,81]. Viral mutation rate can also be influenced by the expression of host genes, such as cellular deaminases that promote hypermutation in RNA viruses [82–84].

Interestingly, the isofemale line that displayed the lowest level of DENV genetic diversity in the midgut (i.e., line CD) was also associated with the highest prevalence and highest viral load in salivary glands. Because we did not examine viral populations in saliva samples, whether this translates in differences of virus transmission potential is unknown. It is tempting to speculate that the vector competence phenotype relates to the level of viral genetic diversity. Unfortunately, we could not compare DENV intra-host diversity in salivary glands between mosquito isofemale lines because DENV amplification was unsuccessful in two out of three lines due to low template concentration. A recent study found differences in the intra-host genetic diversity of West Nile virus among different species of *Culex* mosquitoes [85]. Here, we provided evidence that such differences exist at the intra-specific level. The potential relationship between viral intra-host genetic diversity and vector competence variation among mosquito genotypes deserves further investigation. It will be interesting to determine in future experiments whether the effect of the vector genotype varies according to the mosquito generation, the virus strain, and/or the specific combinations of mosquito lines and virus strains.

Finally, we introduced a non-exclusive, alternative scenario to the ‘diversification’ hypothesis that may contribute to explain why the level of arboviral genetic diversity increases despite a population bottleneck. Our proposed scenario is based on the counter-intuitive idea that a strong initial population bottleneck may actually result in a higher observed level of genetic diversity if low-frequency SNVs go undetected for methodological reasons. We used a model based on stochastic simulations to illustrate the effect of a minimum detection threshold of low-frequency SNVs on observed genetic diversity. When all SNVs present were detected regardless of their frequency (i.e., no detection threshold), mean viral genetic diversity indices decreased following a simulated population bottleneck. Conversely, mean genetic diversity indices increased when only SNVs present at a frequency >1% were successfully detected. In our empirical data, it was not possible to ascertain whether SNVs newly detected after the initial population bottleneck resulted from *de novo* mutations or were already present prior to the bottleneck at frequencies lower than the detection threshold. However, our model indicated that a change in the SNV frequency spectrum following the population bottleneck combined with a minimum detection threshold is a potential explanation to the observed increased genetic diversity following the initial bottleneck.

Taken together, our results show that DENV intra-host genetic diversity in the mosquito vector is shaped by stochastic events during initial midgut infection due to a sharp reduction in population size, followed by predominantly purifying selection during population expansion and diversification in the midgut. Differential diversification between mosquito isofemale lines indicates a genetic foundation, but the lack of convergent SNVs does not support the existence of mosquito genotype-specific directional selection. We conclude that the evolution of DENV intra-host genetic diversity in mosquitoes is not only driven by genetic drift and purifying selection, but is also modulated by vector genetic factors. Characterizing the evolutionary forces that govern arboviral genetic diversity contributes to understanding their unique biology and adaptive potential.

## Supporting Information

S1 Fig Sequencing coverage and depth by sample. (PDF)

S2 Fig Distribution of SNV positions and their mean detected frequencies in the conservative marker set. (A) Bars represent the density of markers retained in the conservative marker set for diversity and natural selection analyses along the DENV reference genome indicated on the *x*-axis. (B) Each dot represents the minor allele frequency of a single SNV along the DENV reference genome indicated on the *x*-axis, averaged over all samples from the same time point in which the SNV was detected. Dot size corresponds to the number of samples from the same time point in which the SNV was detected. The horizontal red dashed line represents a frequency of 0.5 above which a new variant becomes the consensus sequence. SNV distributions are stratified by time point. C = capsid protein, E = envelope glycoprotein, M = membrane glycoprotein, NS1 = non-structural glycoprotein 1; NS2A = non-structural protein 2A; NS2B = non-structural protein 2B; NS3 = non-structural protein 3 (protease/helicase); NS4A = non-structural protein 4A; NS4B = non-structural protein 4B; NS5 = non-structural protein 5 (RNA-dependent RNA polymerase).
(PDF)

S3 Fig Distribution of SNV positions and their mean detected frequencies in the full marker set. Each dot represents the minor allele frequency of a single SNV along the DENV reference genome indicated on the *x*-axis, averaged over all samples from the same time point and isofemale line in which the SNV was detected. Dot size corresponds to the number of samples from the same time point and isofemale line in which the SNV was detected. The horizontal red dashed line represents a frequency of 0.5 above which a new variant becomes the consensus sequence. SNV distributions are stratified by time point and isofemale line. C = capsid protein, E = envelope glycoprotein, M = membrane glycoprotein, NS1 = non-structural glycoprotein 1; NS2A = non-structural protein 2A; NS2B = non-structural protein 2B; NS3 = non-structural protein 3 (protease/helicase); NS4A = non-structural protein 4A; NS4B = non-structural protein 4B; NS5 = non-structural protein 5 (RNA-dependent RNA polymerase).
(PDF)

S4 Fig Proportion of variable sites detected in the full marker set. Letters indicate statistically significant pairwise differences between time points.
(PDF)

S5 Fig Observed levels of DENV intra-host genetic diversity using the conservative marker set. (A) Proportion of variable sites detected. (B) Averaged Shannon entropy (*S*_*n*_) per site over all positions per sample. (C) Averaged nucleotide diversity (*π*) over all positions per sample;
(PDF)

S1 Table *d*_*N*_/*d*_*S*_ ratios using the conservative marker set. (DOCX)

S1 File Primers used for mosquito RAD sequencing and virus deep sequencing. (XLSX)

S2 File R script used for bottleneck simulations. (TXT)
