# Supplementary figures and images for: Genetic Drift, Purifying Selection and Vector Genotype Shape Dengue Virus Intra-host Genetic Diversity in Mosquitoes
Source: PLoS Genet. 2016 Jun 15;12(6):e1006111. doi: 10.1371/journal.pgen.1006111 (PMC4909269; doi:10.1371/journal.pgen.1006111)

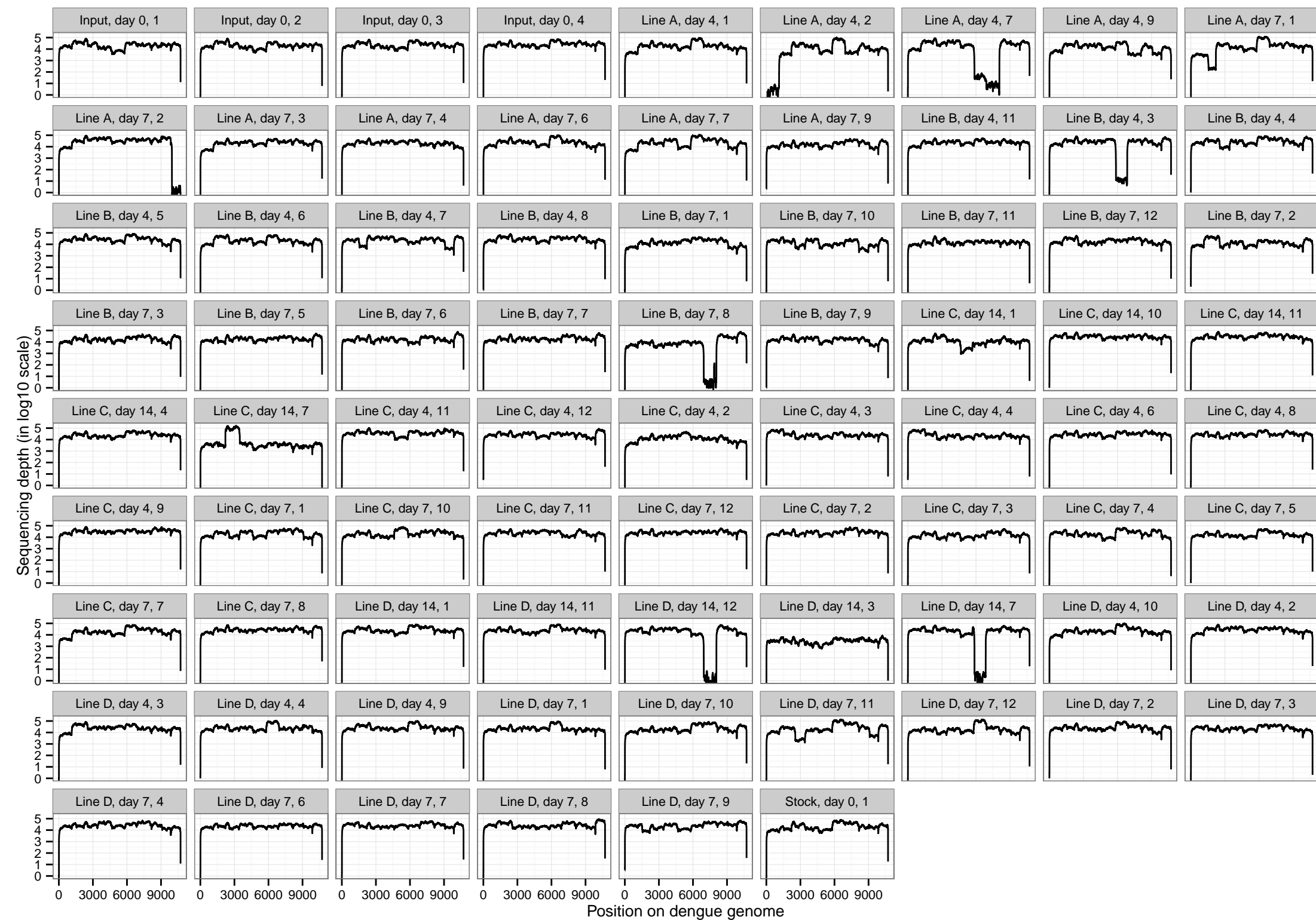

Supplement: S1 Fig — (PDF) [file pgen.1006111.s001.pdf]

A

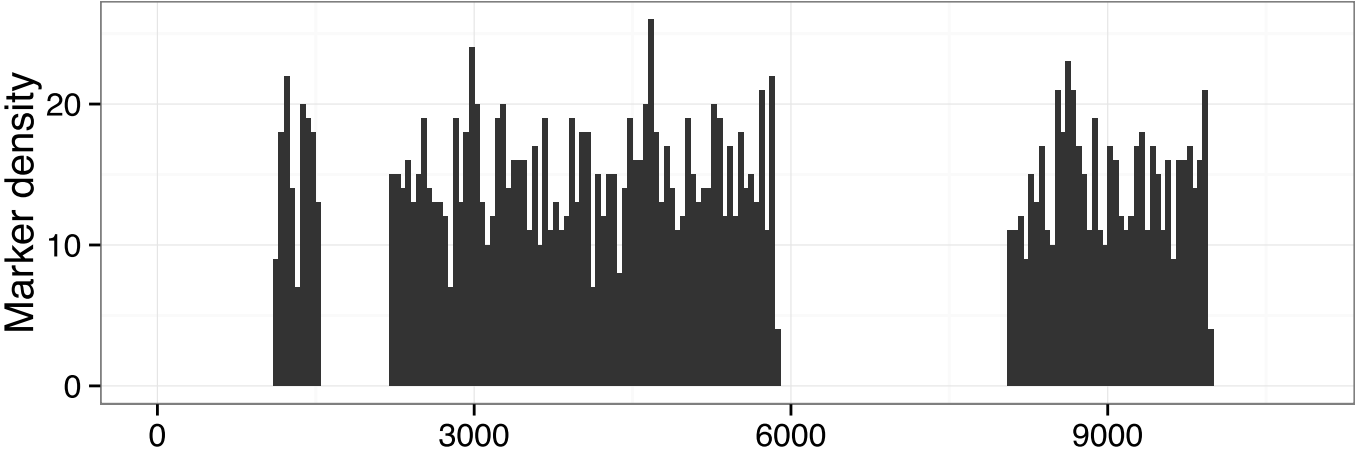

B

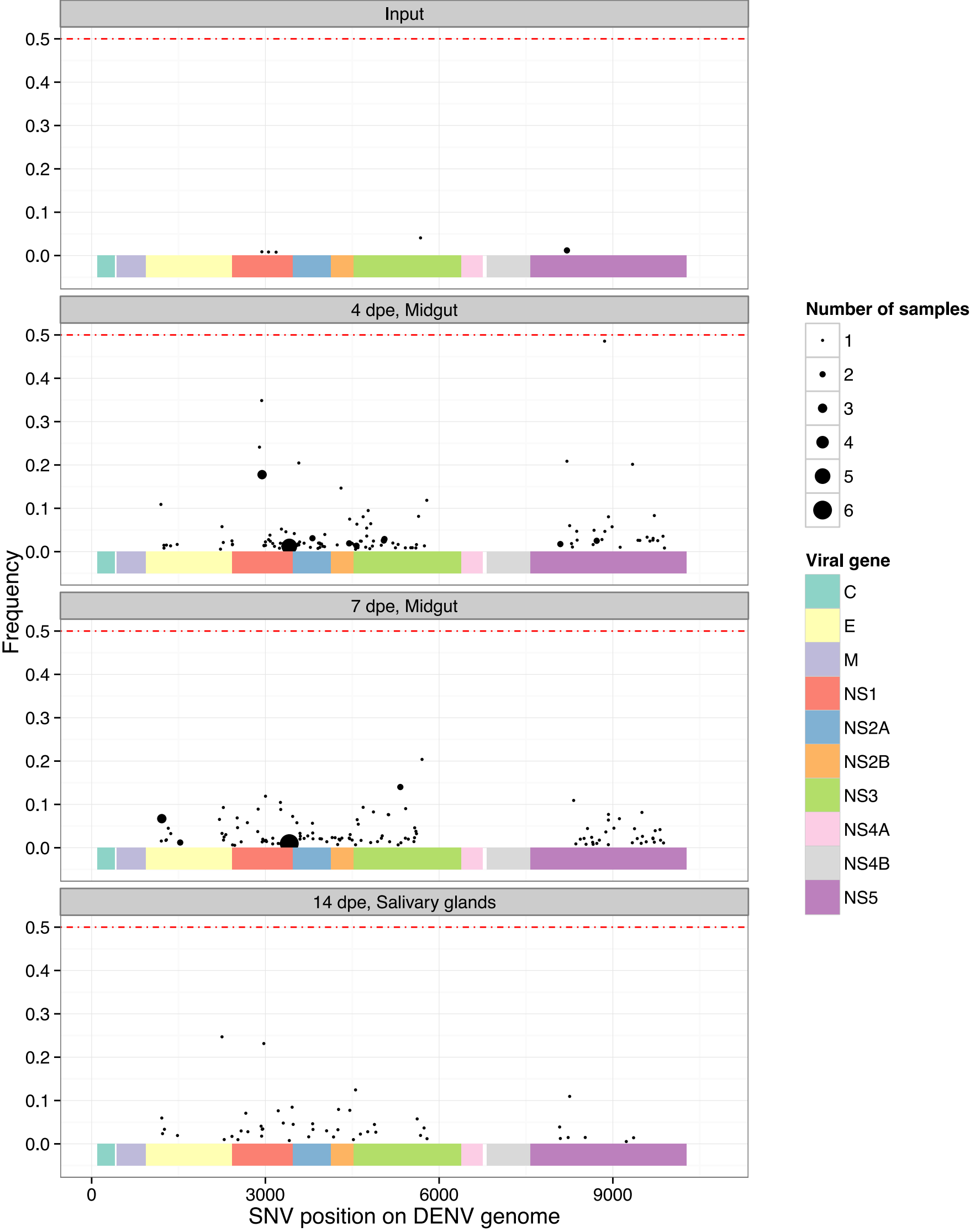

Supplement: S2 Fig — (A) Bars represent the density of markers retained in the conservative marker set for diversity and natural selection analyses along the DENV reference genome indicated on the x-axis. (B) Each dot represents the minor allele frequency of a single SNV along the DENV reference genome indicated on the x-axis, averaged over all samples from the same time point in which the SNV was detected. Dot size corresponds to the number of samples from the same time point in which the SNV was detected. The horizontal red dashed line represents a frequency of 0.5 above which a new variant becomes the consensus sequence. SNV distributions are stratified by time point. C = capsid protein, E = envelope glycoprotein, M = membrane glycoprotein, NS1 = non-structural glycoprotein 1; NS2A = non-structural protein 2A; NS2B = non-structural protein 2B; NS3 = non-structural protein 3 (protease/helicase); NS4A = non-structural protein 4A; NS4B = non-structural protein 4B; NS5 = non-structural protein 5 (RNA-dependent RNA polymerase). (PDF) [file pgen.1006111.s002.pdf]

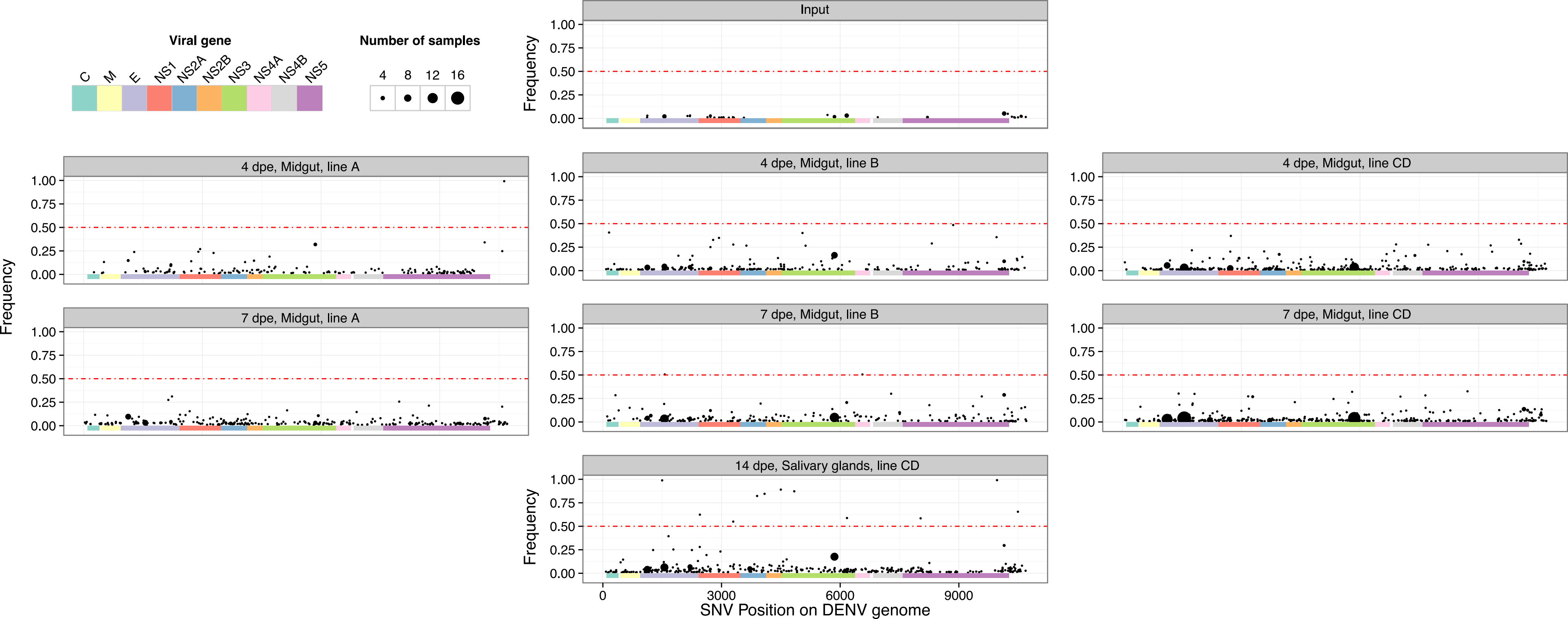

Supplement: S3 Fig — Each dot represents the minor allele frequency of a single SNV along the DENV reference genome indicated on the x-axis, averaged over all samples from the same time point and isofemale line in which the SNV was detected. Dot size corresponds to the number of samples from the same time point and isofemale line in which the SNV was detected. The horizontal red dashed line represents a frequency of 0.5 above which a new variant becomes the consensus sequence. SNV distributions are stratified by time point and isofemale line. C = capsid protein, E = envelope glycoprotein, M = membrane glycoprotein, NS1 = non-structural glycoprotein 1; NS2A = non-structural protein 2A; NS2B = non-structural protein 2B; NS3 = non-structural protein 3 (protease/helicase); NS4A = non-structural protein 4A; NS4B = non-structural protein 4B; NS5 = non-structural protein 5 (RNA-dependent RNA polymerase). (PDF) [file pgen.1006111.s003.pdf]

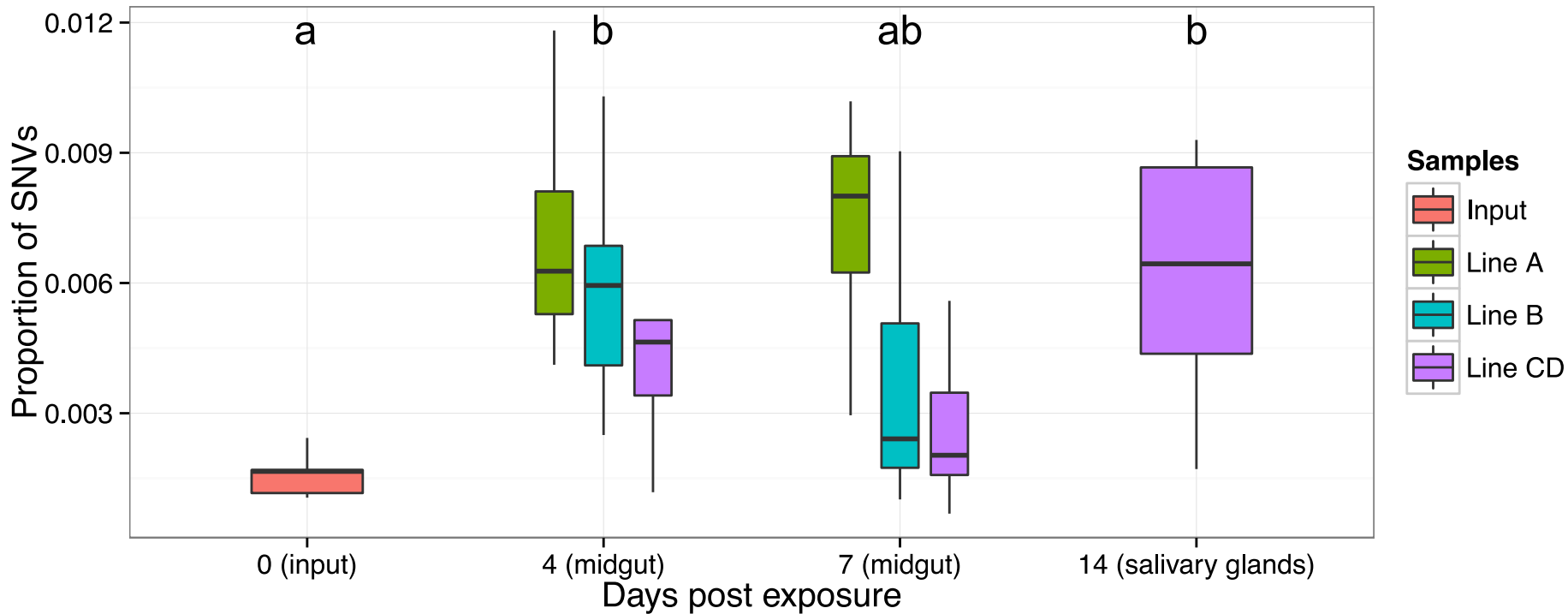

Supplement: S4 Fig — Letters indicate statistically significant pairwise differences between time points. (PDF) [file pgen.1006111.s004.pdf]

A

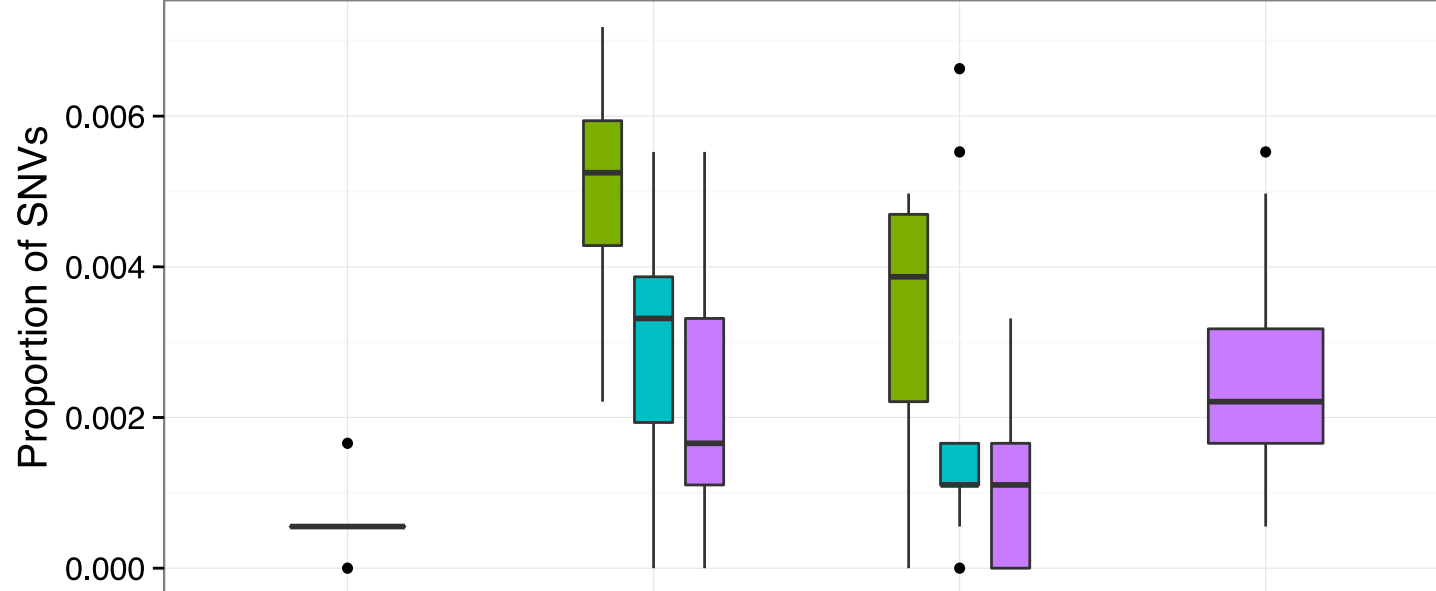

B

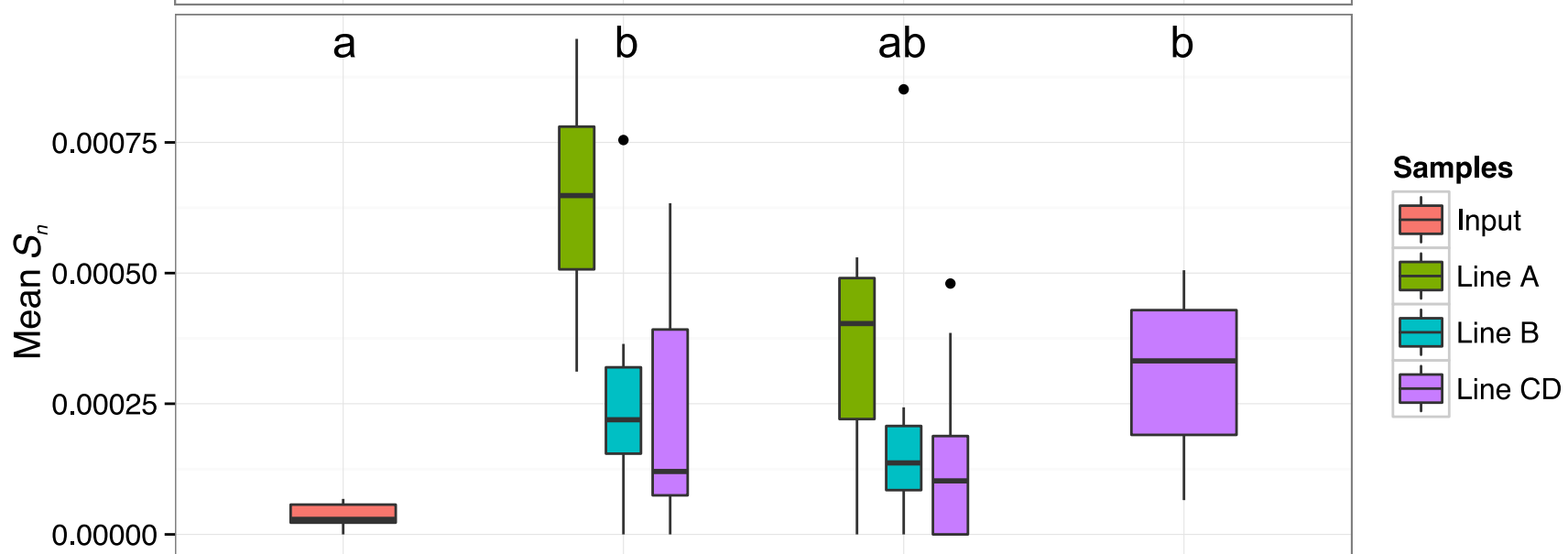

C

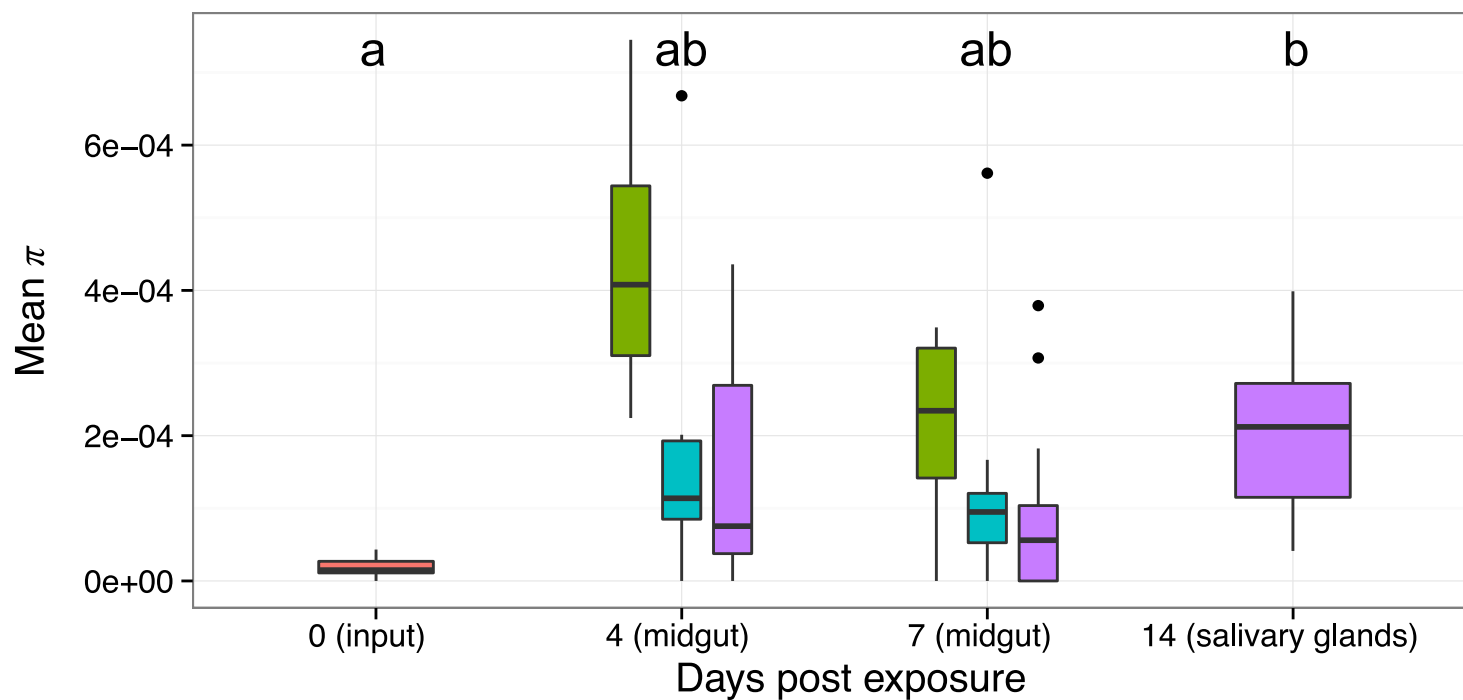

Supplement: S5 Fig — (A) Proportion of variable sites detected. (B) Averaged Shannon entropy (Sn) per site over all positions per sample. (C) Averaged nucleotide diversity (π) over all positions per sample; (PDF) [file pgen.1006111.s005.pdf]
