# Supplementary material for: Genetic Drift, Purifying Selection and Vector Genotype Shape Dengue Virus Intra-host Genetic Diversity in Mosquitoes
Source: PLoS Genet. 2016 Jun 15;12(6):e1006111. doi: 10.1371/journal.pgen.1006111 (PMC4909269; doi:10.1371/journal.pgen.1006111)
Supplement: S1 Table — (DOCX) [file pgen.1006111.s006.docx]

**S1 Table:** ***d_N_/d_S_* ratios using the conservative marker set.**

| **Day post exposure** | **Sample** | ***d_N_*** | ***d_S_*** | ***d_N_*/*d_S_*** |
| --- | --- | --- | --- | --- |
| 0 | Input | 5.503035×10^-6^ | 2.480305×10^-5^ | 0.2218693 |
| 4 | Line A | 2.151092×10^-4^ | 9.700066×10^-4^ | 0.2217606 |
|  | Line B | 1.033578×10^-4^ | 4.659567×10^-4^ | 0.2218186 |
|  | Line CD | 7.363042×10^-5^ | 3.319168×10^-4^ | 0.2218340 |
| 7 | Line A | 1.086174×10^-4^ | 4.896736×10^-4^ | 0.2218158 |
|  | Line B | 4.520744×10^-5^ | 2.037760×10^-4^ | 0.2218487 |
|  | Line CD | 1.538954×10^-5^ | 6.936470×10^-5^ | 0.2218642 |
| 14 | Line CD | 1.174938×10^-4^ | 5.297019×10^-4^ | 0.2218112 |
